# New MgFeAl-LDH Catalysts for Claisen–Schmidt Condensation

**DOI:** 10.3390/molecules27238391

**Published:** 2022-12-01

**Authors:** Rodica Zăvoianu, Mădălina Tudorache, Vasile I. Parvulescu, Bogdan Cojocaru, Octavian D. Pavel

**Affiliations:** 1 Faculty of Chemistry, University of Bucharest, 4-12 Regina Elisabeta Avenue, 030018 Bucharest, Romania; 2 Research Center for Catalysts & Catalytic Processes, Faculty of Chemistry, University of Bucharest, 4-12 Regina Elisabeta Avenue, S3, 030018 Bucharest, Romania

**Keywords:** MgFeAl-LDH, mechano-chemical, tetramethylammonium hydroxide, Claisen–Schmidt condensation

## Abstract

A rapid, cheap and feasible new approach was used to synthesize the Mg_0.375_Fe_0.375_Al_0.25_-LDH in the presence of tetramethylammonium hydroxide (TMAH), as a nontraditional hydrolysis agent, applying both mechano-chemical (MC) and co-precipitation methods (CP). For comparison, these catalysts were also synthesized using traditional inorganic alkalis. The mechano-chemical method brings several advantages since the number of steps and the energy involved are smaller than in the co-precipitation method, while the use of organic alkalis eliminates the possibility of contaminating the final solid with alkaline cations. The memory effect was also investigated. XRD studies showed Fe_3_O_4_ as stable phase in all solids. Regardless of the alkalis and synthesis methods used, the basicity of catalysts followed the trend: mixed oxides > parent LDH > hydrated LDH. The catalytic activity of the catalysts in the Claisen–Schmidt condensation between benzaldehyde and cyclohexanone showed a linear dependence to the basicity values. After 2 h, the calcined sample cLDH-CO_3_^2−^/OH^−^-CP provided a conversion value of 93% with a total selectivity toward 2,6-dibenzylidenecyclohexanone. The presence of these catalysts in the reaction media inhibited the oxidation of benzaldehyde to benzoic acid. Meanwhile, for the self-condensation of cyclohexanone, the conversions to *mono*- and *di*-condensed compounds did not exceed 3.8%.

## 1. Introduction

Layered double hydroxides (LDHs) are a class of ionic solids characterized by a layered structure with a generic layer sequence showing a general formula **[M^2+^_1−x_M^3+^_x_(OH)_2_]^x+^[A^n−^_x/n_]·mH_2_O** [1], with ***M^2+^*** and ***M^3+^*** as bi- and trivalent cations in an octahedral geometry [2], ***A*** as an anion with charge ***n***, ***x*** as the M^3+^/(M^2+^ + M^3+^) ratio and ***m*** as the number of water molecules [3,4]. While these kind of solids were firstly discovered in Sweden in mineral ores around 1842, their first artificial synthesis was covered one century later by a patent in 1970 [5]. However, the real interest in this class of solids with base properties started with the work of Cavani et al. [1]. It has been strengthened by potential applications in different areas of strategic importance such as medicine [6], environmental protection, ion exchange, adsorption [7,8], polymer stabilization [9,10,11], corrosion inhibition [12], catalysis [13,14], batteries [15], filtration [16], etc.

Such materials are constituted from positively charged brucite-like layers with negatively charged interlayers compensating anions as well as water molecules, resulting in a charge-balanced architecture [17]. These structures can incorporate a large number of cations with dimensions close to those of Mg^2+^, such as Ni^2+^, Cu^2+^, Zn^2+^, Co^2+^, Fe^2+^, Mn^2+^, Ga^3+^, Fe^3+^, Al^3+^, In^3+^, Ti^4+^, Zn^4+^ [1,18], etc. Thus, depending on the number of the cations, LDHs with binary [19], ternary [20], or quaternary [21] structures can be produced. Further tuning of the particular electronegativity of the cations may lead to the optimization of the acid-base properties (ditopic character) of the synthesized materials. Furthermore, due to the flexibility of the interlayer space, larger anions such as phthalocyanines [22], polyoxometalate [23], citrate [24], etc. can be also inserted in the structure. Even more, recent publications reported the intercalation of large structures such as graphene [25], carbon nanotubes [26], polymers [27], etc., opening opportunities to more complex materials.

Traditionally, the LDH can be synthesized by several methods [1] such as (i) co-precipitation at constant pH, at low/high supersaturation, or increasing pH, by titration with NaOH and/or NaHCO_3_; (ii) hydrothermal treatments under pressure in an autoclave at a higher temperature or by aging at a lower temperature; and (iii) anionic exchange or memory effect reconstruction, which enables the reformation of the layered structure following the hydration of the mixed oxides generated by thermal treatments applied to the parent LDH at temperatures that do not exceed 600 °C. However, these methods have some disadvantages such as numerous steps, high energy, time consumption, large volumes of water for washing the solids, etc. Other routes such as urea hydrolysis, sol-gel, electro-synthesis or the mechano-chemical method may offer alternatives for the preparation of these materials. Even though, the ball milling in the absence of water or other solvents [28,29,30,31] has some limitations. Larger cations such as La (1.032 Å) or Y (0.9 Å) [18] can be accommodated into the octahedral positions of the layered network only in small amounts.

The use of organic alkalis seems to be an extremely good alternative to these drawbacks despite a higher price compared to that of the inorganic ones. Several LDH materials containing larger cations such as Cu/Mn-LDH [32], Zn/Al-LDH [33], Ni/Cr, Ni/Fe, or Ni/Co-LDHs [34] were prepared in this way. Our group also investigated LDH syntheses in the presence of organic alkali focusing on the size of the chain [35].

Among the investigated reactions in the presence of the LDH catalysts [36], aldol condensation, Claisen–Schmidt and Knoevenagel reactions, Michael addition, Baeyer–Villiger oxidation, Weitz–Scheffer epoxidation, nitriles hydrolysis, alkylation, and acylation or polymerization are most frequently considered as references. Specifically, Claisen–Schmidt condensation [37,38] is dedicated to the formation of new C–C bonds. The condensation of cyclohexanone and benzaldehyde to *mono*-, 2-benzylidene-cyclohexanone, or *di*-condensation, 2,6-dibenzylidene-cyclohexanone, is also an important subject [39,40,41,42,43,44,45,46]. However, the Claisen–Schmidt condensation has some limitations due to the possibility of the appearance of side reactions, such as self-condensation, as an effect of the additional presence of the acid sites.

Already published articles showed that for this reaction, the conversions may reach up to 60% in the presence of mixed oxide type catalysts with pronounced basicity obtained from layered materials modified with rare-earth cations [47]. The same activity was also displayed by a perfluorosulfonic acid resin catalyst, HRF5015 [48]. On the other hand, benzaldehyde may be oxidized with oxygen from air, both in the absence/presence of the catalyst. In that case the performances are related to the acidity strength of the catalyst [49].

Starting from this state of the art, the aims of this paper are: (i) to bring new perspectives in adapting the physicochemical properties of Mg_0.325_Fe_0.325_Al_0.25_-LDH through two non-traditional parameters, e.g., the mechano-chemical preparation method and the use of organic alkali (TMAH) as a hydrolysis agent; (ii) the possibility of protecting Fe^2+^ against oxidation by its inclusion in a solid LDH network; (iii) to optimize the Claisen–Schmidt condensation between benzaldehyde and cyclohexanone to the desired condensation product.

## 2. Results and Discussion

### 2.1. Characterization of Catalysts

**Figure 1:**
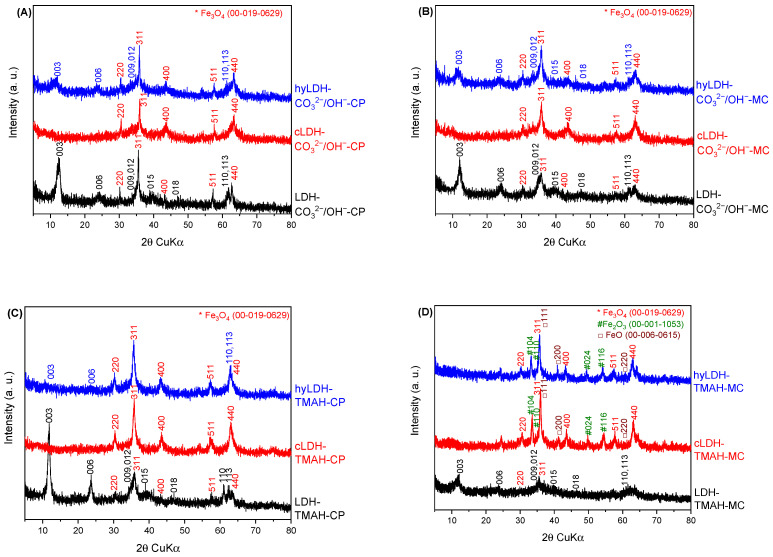
XRD patterns of the synthesized materials (**A**)—co-precipitation method/inorganic alkali; (**B**)—mechano-chemical method/inorganic alkali; (**C**)—co-precipitation method/organic alkali; (**D**)—mechano-chemical method/organic alkali; * Fe_3_O_4_; # Fe_2_O_3_; □ FeO).

Except for the LDH-TMAH-MC (Figure 1D, in black) the XRD patterns of the parent LDHs presented the typical reflections of layered materials with intense and symmetric reflections at small angles and broad and weak reflections at higher angles [1].

The presence of iron was identified from the distinguishable lines of Fe_3_O_4_ (ICDD 00-019-0629, intense *311* and weaker *220*, *400*, *511*, and *440*) which highlight the oxidation of Fe^2+^ to Fe^3+^ with the formation of the stable FeO·Fe_2_O_3_ structure. While the network parameter ***a*** (expressing the cation-cation distance in the layered structure) did not change significantly, the ***c*** parameter changed, proving a slight increase for the co-precipitated samples compared to the mechano-chemical ones (Table 1) associated with the presence of small amounts of TMAH and tri-methyl amine (an impurity present in TMAH) [39].

At the same time, the value of ***c*** parameter is slightly higher in the case of mechano-chemically obtained samples compared to those obtained by co-precipitation due to the higher amount of embedded Fe_3_O_4_ phase. It should be highlighted that the pattern with the most intense diffraction lines is that of the sample obtained in the presence of TMAH, i.e., LDH-TMAH-CP, which also has the lowest value of crystallite size (111 Å). The LDH-TMAH-MC sample contains a high amount of amorphous phase with the largest dimension of crystallite size of 600 Å. The impurity phases, obtained during the synthesis, are probably located in the interlayer space as well as onto the surface of the LDH lamellae, as is suggested by the decreasing of the I*_003_*/I*_110_* ratio value, e.g., 4.30 (LDH-CO_3_^2−^/OH^−^-CP); 3.50 (LDH-CO_3_^2−^/OH^−^-MC); 3.73 (LDH-TMAH-CP); 1.13 (LDH-TMAH-MC) as well as by the shift to lower 2θ values of the *003* diffraction lines (12.6607; 11.9400; 11.7720; 11.2666) [50]. The calcination of LDH samples at 460 °C for 18 h lead to corresponding mixed oxides where Fe_3_O_4_ is the prevailing crystalline phase, Figure 1 (red lines). Using TMAH in the mechano-chemical preparation generated more crystalline phases of Fe_x_O_y_, e.g., FeO (ICDD 00-006-0615), Fe_2_O_3_ (ICDD 00-001-1053) and Fe_3_O_4_ (ICDD 00-019-0629). The presence of these separate oxides in the calcined samples may be a consequence of the co-existence of Fe^2+^ and Fe^3+^ amorphous hydroxyl/hydroxycarbonate phases in the parent LDH material. Therefore, it can be concluded that part of Fe^2+^ is oxidized to Fe^3+^ and the presence of the organic alkali does not succeed in avoiding the oxidation.

The reconstruction of the layered structure by memory effect occurs slightly for the materials prepared with inorganic alkalis, while in the case of the materials synthesized in the presence of TMAH the diffraction lines corresponding to the LDH structure have a very low intensity. The I*_003_*/I*_006_* ratio shows a sudden decrease from the parent LDHs to the rehydrated samples, a fact that sustains the above statement.

The presence of stable oxides in the rehydrated samples is also highlighted by the increased values of the IFS parameter for the LDH phase for the samples hyLDH-CO_3_^2−^/OH^−^-CP, hyLDH-CO_3_^2−^/OH^−^-MC, and hyLDH-TMAH-CP, compared to the corresponding parent LDHs. Both cLDH-TMAH-MC and hyLDH-TMAH-MC present similar diffraction lines, suggesting a higher stability of the oxide phases and, as a consequence, a poor memory effect. Meanwhile, for the sample hyLDH-TMAH-MC that has the largest crystallite size D*_003_* (1296 Å), the IFS parameter decreases to 2.63 compared to the 2.84 of LDH-TMAH-MC. This fact may be a consequence of the formation of a compact mixture of oxide phases.

**Figure 2:**
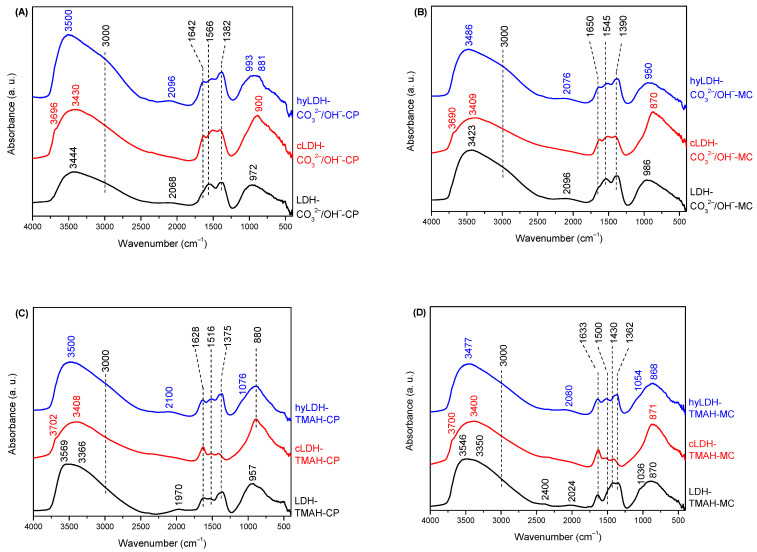
DRIFT spectra of the materials ((**A**)—co-precipitation method/inorganic alkali; (**B**)—mechano-chemical method/inorganic alkali; (**C**)—co-precipitation method/organic alkali; (**D**)—mechano-chemical method/organic alkali).

Diffuse reflectance infrared Fourier transform (DRIFT) spectra of the parent LDH (Figure 2, black line) present a large band in the 3700–3400 cm^−1^ domain corresponding to the vibration of hydroxyl groups, υO-H, while that at 3000 cm^−1^ is assigned to hydrogen bonds present between carbonate anion and water molecules from the interlayer space [29].

The band at 1628–1650 cm^−1^ is characteristic to the H_2_O bending vibration in the LDH interlayer, while the one at 1500–1566 cm^−1^ is assigned to C=O bond from the surface. The characteristic vibrations for bidentate carbonate ν(CO_3_^2−^) are noticed at 1362–1390 cm^−1^, and 1200–600 cm^−1^. The bands below 600 cm^−1^ correspond to Mg–O and Al–O bonds while the one at 450–460 cm^−1^ is characteristic to Fe-O vibrations. The calcined samples show a new band at 3690–3702 cm^−1^ characteristic to the stretching vibrations of isolated structural hydroxyl groups connected to hexacoordinated Mg species [52] highly dispersed in the solid matrix. The presence of an inflexion point around 3000 cm^−1^ in the spectra of the calcined samples prepared in the presence of inorganic alkalis is due to the residual CO_3_^2−^ and OH^−^ after the calcination at a temperature of 460 °C. Noteworthy, this temperature is the key factor responsible for the memory effect. Higher temperatures lead to the stabilization of the cations in the tetrahedral positions [53].

As an interesting fact, only the LDH-TMAH-CP sample showed a very weak band characteristic to Fe^0^-CO at 1970 cm^−1^ stretching [54]. The samples prepared in the presence of inorganic alkalis presented bands at 2068 and 2096 cm^−1^, respectively, attributed to Fe^2+^-CO stretching vibration. Hence it may be inferred that iron carbonic phases are present in the parent LDH under an amorphous state and are not visible in the XRD patterns. It should be also highlighted that regardless of the type of LDH, Fe^2+^ is oxidized to Fe^3+^ during the hydration process, as it is evidenced by the presence of the weak bands at 2011–2076 cm^−1^ corresponding to the Fe^3+^-CO stretching [54].

**Figure 3:**
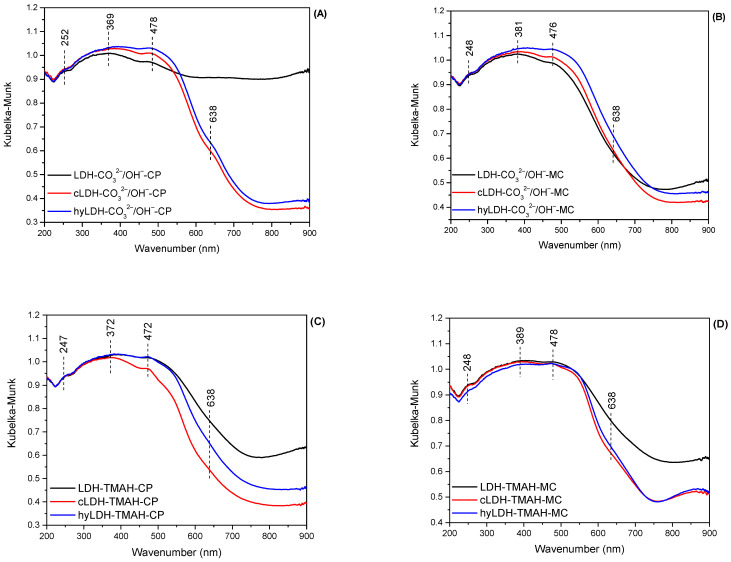
**DR**-UV-Vis spectra of the materials ((**A**)—co-precipitation method/inorganic alkali; (**B**)—mechano-chemical method/inorganic alkali; (**C**)—co-precipitation method/organic alkali; (**D**)—mechano-chemical method/organic alkali).

The DR-UV-Vis spectra of the samples (Figure 3) display a quasi-similar shape, proving the simultaneous presence of Fe^2+^ and Fe^3+^ species. The band at 638 nm corresponding to the Fe^2+^ species is more intense for the fresh sample prepared using the co-precipitation method with inorganic alkali. It has similar intensities for the fresh samples prepared with TMAH, and the lowest intensity for the fresh LDH-CO_3_^2−^/OH^−^-MC, most probably caused by the oxidation during the mechano-chemical process. It should be noted that the fresh sample prepared with TMAH by the mechano-chemical method was not oxidized to the same extent as the one prepared with inorganic alkali. The intensity of the absorbance in this region decreased significantly for the calcined and rehydrated samples, confirming the oxidation of Fe^2+^ species during these treatments.

All the spectra showed strong absorption below 500 nm due to the presence of Fe^3+^ species [55]. However, several specific species were also distinguished from the bands at 247–252 nm assigned to isolated Fe^3+^, at 369–389 nm characteristic to Fe_x_O_y_ oligomers, and at 472–478 nm assigned to Fe_2_O_3_ particles [56].

**Figure 4:**
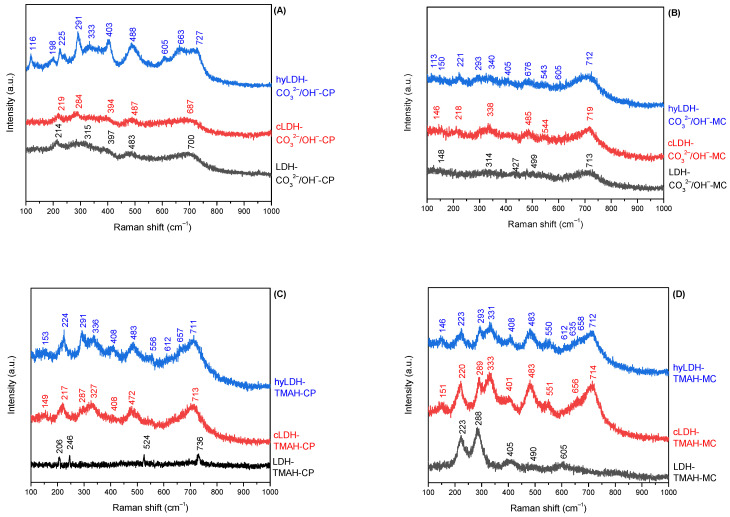
Raman spectra of the materials ((**A**)—co-precipitation method/inorganic alkali; (**B**)—mechano-chemical method/inorganic alkali; (**C**)—co-precipitation method/organic alkali; (**D**)—mechano-chemical method/organic alkali).

The characterization by Raman spectroscopy (Figure 4) confirmed the presence of the iron oxides even in the parent LDH samples, a fact also certified by the above presented characterization techniques. Thus, the characteristic bands of hematite Fe_3_O_4_, undergoing a slight shifting depending on the spatial disposition were observed (E_1g_ 200–247 cm^−1^; E_1g_ 291–293 cm^−1^; E_1g_ 315–336 cm^−1^; E_1g_ 403–408 cm^−1^; A_1g_ 483–488 cm^−1^; E_1g_ 605–612 cm^−1^) [57]. The calcined/hydrated mechano-chemically synthesized samples presented weak bands corresponding to maghemite (γ-Fe_2_O_3_) at 340 cm^−1^ (T_1_), 483–485 cm^−1^ (E), and 700–730 cm^−1^ (A_1_). This fact is somewhat unexpected because in accordance to the literature data at 400 °C, i.e., below the calcination temperature (460 °C), the Fe_3_O_4_ phase usually turns into α-Fe_2_O_3_, while the γ-Fe_2_O_3_ phase is stable in the temperature range of 200–400 °C [57]. However, the variable intensity ratio of E_1g_ 200–247 cm^−1^ and E_1g_ 291–293 cm^−1^ levels indicated the presence of a certain heterogeneity at the micro-scale level of the iron mixed oxide samples [58]. A weak band at 656–663 cm^−1^ corresponding to FeO (wustite) was also observed for the calcined/hydrated samples [59].

Regardless of the preparation route or the base, the basicity of the iron-containing samples (Table 2) presented an atypical variation, i.e., mixed oxides > parent LDH > reconstructed LDH, also showing the lowest value for the hydrated samples. It was also different to Mg(M^II^)/Al(M^III^)-LDH [3,29,39] where the trend was mixed oxides > reconstructed LDH > parent LDH. This behavior can be assigned to the presence of the iron oxides in the layered materials (parent or hydrated). Nevertheless, a certain influence of the CO_3_^2−^/OH^−^ ratio cannot be totally eliminated, since these are present in the hydrated materials prepared by the co-precipitation with inorganic alkalis. We can therefore speculate that the lower basicity of the hydrated LDH compared to the parent LDH is due to the structural alterations of the oxides in the presence of water. These alterations were already evidenced by the characterization techniques.

**Figure 5:**
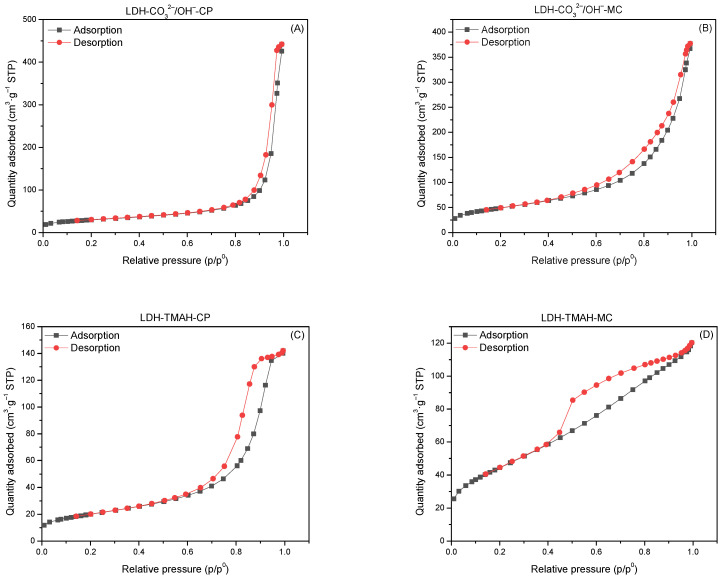
The adsorption–desorption isotherms of LDH sample.

All the samples showed a Type IV adsorption–desorption isotherm with a shape characteristic to mesoporous materials (Figure 5). Only LDH-TMAH-MC showed a deviation from the traditional shape due to the presence of a mixture of amorphous phases.

**Figure 6:**
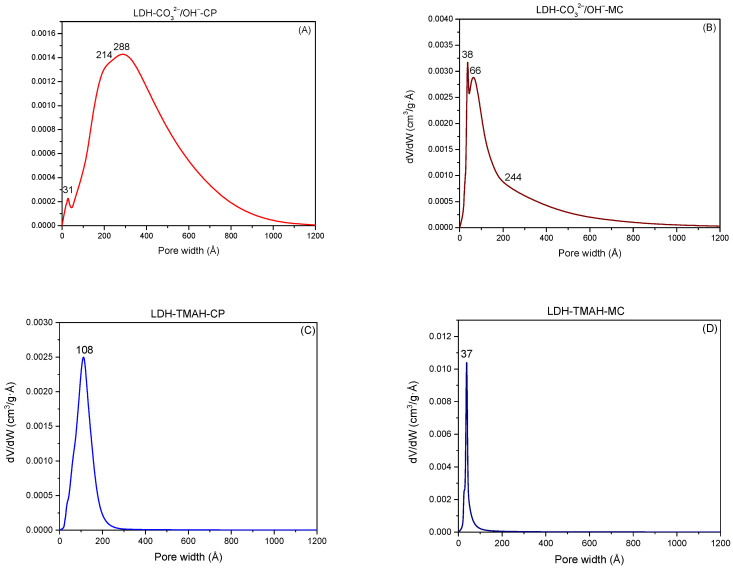
The representation of LDH sample pore widths.

In addition to the hydrolysis agent activity, TMAH also behaves in the synthesis of LDH as a template molecule that generates a porosity that does not exceed the value of 200 Å, while inorganic alkalis generate porosity on a wide range up to 1200 Å (Figure 6). If in the case of mechanochemically obtained materials there is only one domain of pores of 108 Å for LDH-TMAH-CP and, respectively, 37 Å for LDH-TMAH-MC, the co-precipitation method leads to obtaining several domains, e.g., 31 Å, 214 Å, and 288 Å for LDH-CO_3_^2−^/OH^−^-CP and 38 Å, 66 Å, and 244 Å for LDH-CO_3_^2−^/OH^−^-MC. Unlike these, LDH-TMAH-MC showed an extremely narrow pore distribution that was even narrower compared to LDH-TMAH-CP.

### 2.2. Catalytic Activity

#### 2.2.1. Claisen–Schmidt Condensation

**Figure 7:**
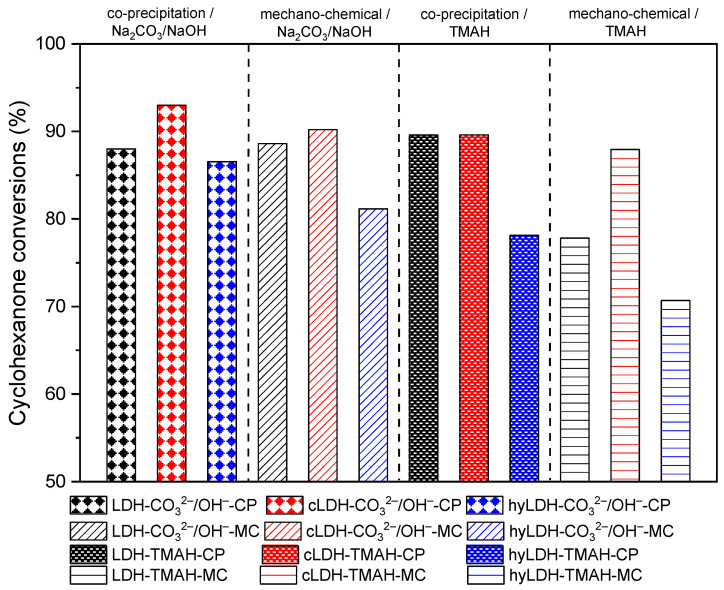
Cyclohexanone conversion of samples (2 h, 120 °C, 20 mg of catalyst, solvent-free).

**Figure 8:**
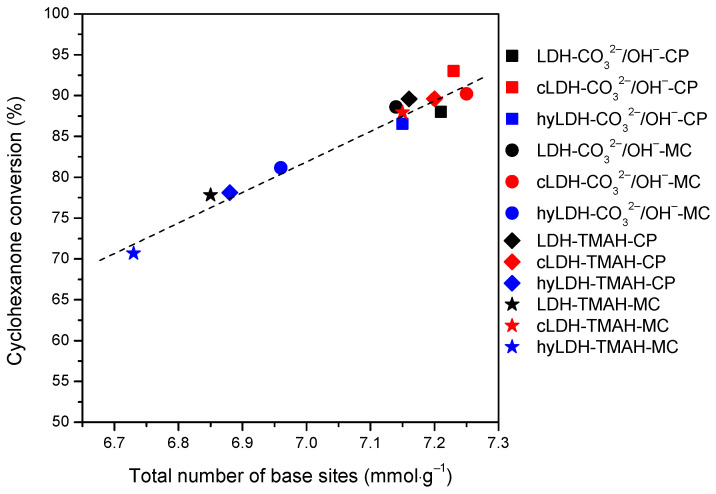
The cyclohexanone conversion vs. total number of base sites for investigated catalysts (2 h, 120 °C, 20 mg of catalyst, solvent-free).

**Scheme 1:**
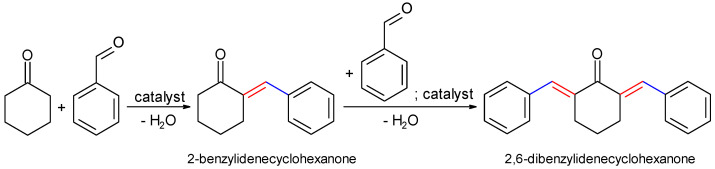
Claisen–Schmidt condensation (benzaldehyde (0.002 mol), cyclohexanone (0.001 mol) and 20 mg of catalyst, 600 rpm, 2 h, 120 °C, solvent-free conditions).

The blank tests at room temperature, after 2h, led to a conversion of cyclohexanone below 1%, that further increased at 10% at 120 °C for a total selectivity to 2,6-dibenzylidene-cyclohexanone (the *di*-condensed product). The samples synthesized by the co-precipitation method showed a higher activity compared to those prepared mechano-chemically (Figure 7). The samples prepared in the presence of TMAH showed slightly lower values compared to those prepared with inorganic alkalis. However, the variation of the catalytic activity kept a linear trend versus the variation of the total basicity, e.g., mixed oxides > parent LDH > hydrated LDH (Figure 8). A total selectivity to the *di*-condensed product has been preserved in all the cases being in accordance to the twisting ability of the *mono*-condensed product. This behavior can also be associated with the possibility of a further adsorption on the surface active sites leading to the *di*-condensed product (Scheme 1) [39]. This assumption is also supported by the cyclohexanone self-condensation where the main product is a *mono*-condensed compound as an effect of a steric hindrance.

Under similar reaction conditions, the investigated mixed oxide catalysts showed superior activities compared to those reported with activated NaOH/fly ash catalyst [41], and comparable to the cesium salts of 12-tungstophosphoric acid catalysts [60] or ultrasound activation [61].

#### 2.2.2. Benzaldehyde Oxidation as Possible Side Reaction

**Scheme 2:**
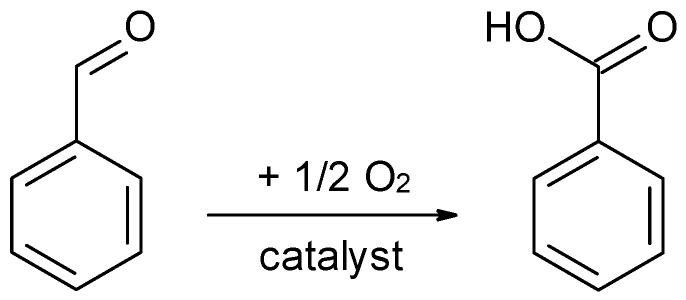
The oxidation of benzaldehyde (0.01 mol benzaldehyde, 53 mg catalyst under solvent-free conditions, 600 rpm, 2 h, 120 °C).

Considering that benzaldehyde can be easily oxidized to benzoic acid, we further investigated the behavior of these catalysts in this oxidation (Scheme 2). The blank test at room temperature after 2 h displays a benzaldehyde conversion of 9%, while after 2 h at 120 °C it increased up to 31.5%. Meanwhile, the presence of these catalysts inhibited the oxidation. As an effect, even the highest conversion was below 6% (cLDH-TMAH-MC), (Table 3). Furthermore, there was no correlation between the catalysts’ basicity and the conversion levels. 

#### 2.2.3. Self-Cyclohexanone Condensation as Possible Side Reaction

**Scheme 3:**
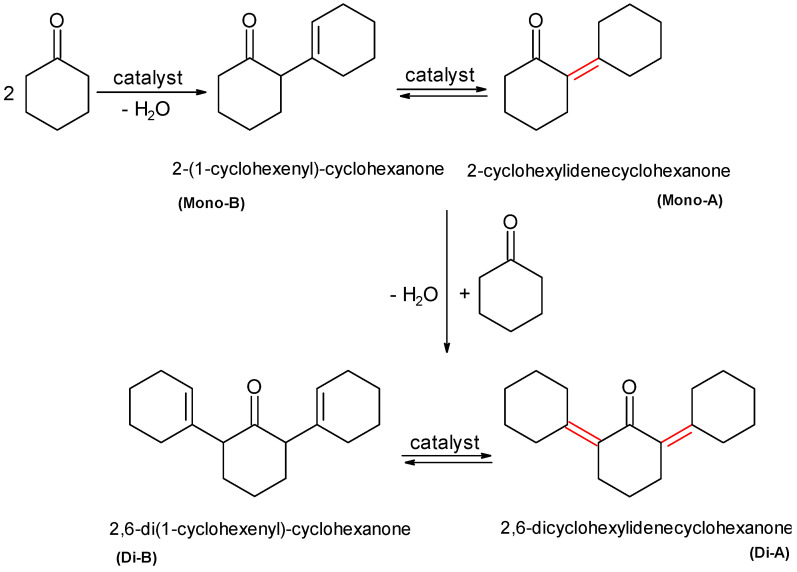
The aldol self-condensation (0.01 mol cyclohexanone, 50 mg catalyst under solvent-free conditions, 600 rpm, 5 h, 120 °C).

The conversion of cyclohexanone in the blank test after 2 h at room temperature did not exceed 1%, while at 120 °C the conversion increased up to 6% with a selectivity of 91.5% to 2-(1-cyclohexenyl)-cyclohexanone (*mono*-condensed product/***Mono-A***). This reaction commonly proceeds via an enolate ion intermediate where the double bonds are able to cyclically migrate between the two cycles generating (***Di-A***) and (***Di-B***) products (Scheme 3) [47].

**Figure 9:**
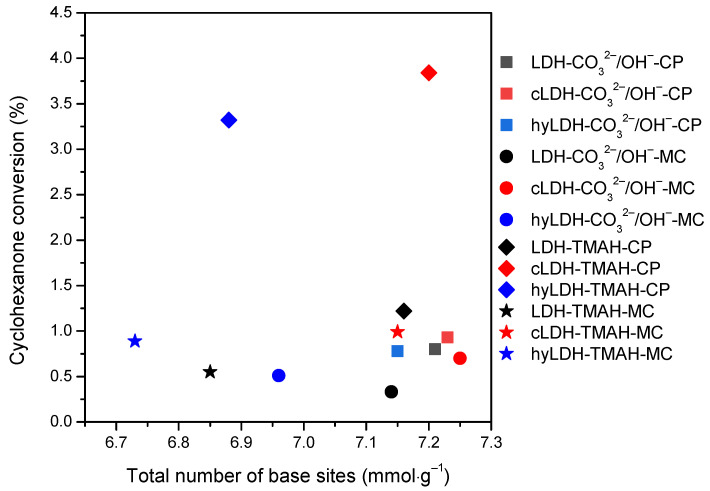
Cyclohexanone conversion vs. total number of base sites of investigated catalysts in self cyclohexanone condensation (2 h, 120 °C, 50 mg catalyst, solvent-free).

Compared to the layered materials, the mixed oxides presented better conversion, but the resulted products were no longer similar to the ones in the Claisen–Schmidt condensation (Figure 9). The variation trend of the conversions on the parent LDHs was also dissimilar to that on the hydrated LDHs. Concerning the selectivity to the *mono*-condensation product, these were superior to those for the *di*-condensed product (Table 4). This certifies the fact that the double bond between the fragments of the *mono*-condensed product (red bond, Scheme 3) stiffens the molecule, preventing its adsorption for a subsequent condensation step. The presence of small amounts of *di*-condensed products is only attributed to the active sites placed on the external geometric surface of the catalysts.

### 2.3. Catalyst Reusability

**Figure 10:**
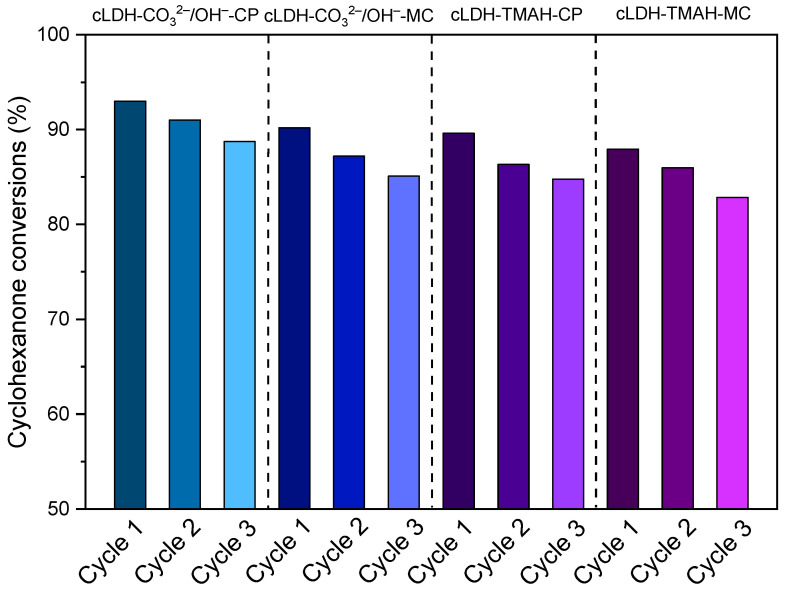
The catalyst reusability after 3 cycles in Claisen–Schmidt condensation for mixed oxides.

**Figure 11:**
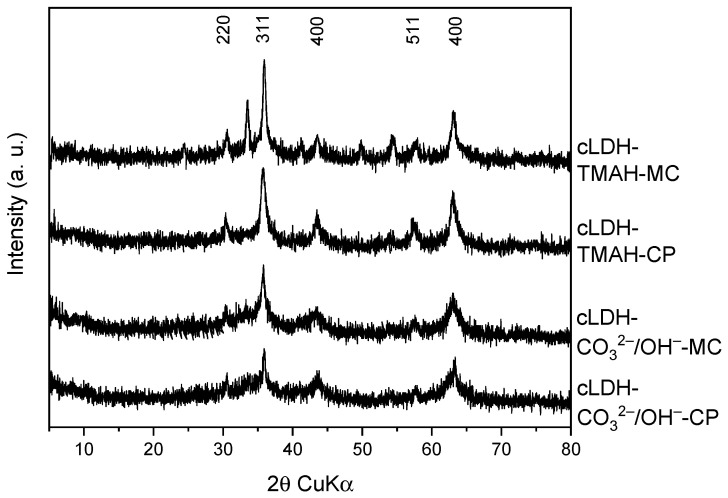
XRD patterns of the reused mixed oxide catalysts after 3 reaction cycles in the Claisen–Schmidt condensation.

The stability of mixed oxides as the best catalysts was checked in several consecutive Claisen–Schmidt condensation runs. After the third cycle, the conversion decreased by less than 5.1% (Figure 10), reaching a plateau level in the fourth and fifth cycle. The stability of the catalysts was confirmed by the fact that the XRD patterns of the solids recovered after the third cycle did not show modifications of the diffraction lines compared to the patterns of the fresh samples (Figure 11).

## 3. Materials and Methods

### 3.1. Catalyst Preparation

The Mg_0.325_Fe_0.325_Al_0.25_-LDH was synthesized using (i) a co-precipitation and (ii) a mechano-chemical method using either inorganic or organic alkalis for the pH adjustment. The co-precipitation was achieved in the presence of inorganic alkalis following a methodology already reported [29,39] by mixing two salt solutions. The first solution contained Mg(NO_3_)_2_·6H_2_O, FeCl_2_·4H_2_O, and Al(NO_3_)_3_·9H_2_O in a 0.325/0.325/0.25 molar ratio at a 1.5 M concentration. The second solution consisted of NaOH and Na_2_CO_3_ at a NaOH/Na_2_CO_3_ = 2.5 molar ratio and 1 M Na_2_CO_3_ concentration. These solutions were mixed at 600 rpm and pH of 10 at RT by using a TIM854, NB pH/EP/Stat pH-STAT Titrator at a feed rate of 60 mL·h^−1^. After the complete precipitation, the suspension was aged for 18 h at 80 °C in an air atmosphere, cooled at room temperature, filtered, washed with bi-distilled water until a pH of 7, and dried for 24 h in air at 120 °C (**LDH-CO_3_^2−^/OH^−^-CP**). The calcination of the resulting solid was carried out at 460 °C for 18 h in air atmosphere leading to the corresponding mixed oxides (**cLDH-CO_3_^2−^/OH^−^-CP**). The reconstruction of the layered structure was carried out through the memory effect by hydration of **cLDH-CO_3_^2−^/OH^−^-CP** with bi-distilled water for 24 h at RT, followed by drying for 24 h at 120 °C in air (**hyLDH-CO_3_^2−^/OH^−^-CP**). The mechano-chemical method was performed by direct milling of the above mentioned precursors in a Mortar Grinder RM 200 for 1 h at 100 rpm (**LDH-CO_3_^2−^/OH^−^-MC**) at a pH close to 10 without the addition of bi-distilled water or submission to an aging process. Further protocols for the synthesis of the corresponding mixed oxides and reconstructed layered samples (**cLDH-CO_3_^2−^/OH^−^-MC** and **hyLDH-CO_3_^2−^/OH^−^-MC**) were identical to the above-described ones. The same co-precipitation and mechano-chemical protocols were also utilized for the synthesis of LDH in the presence of tetramethylammonium hydroxide (wt. 25% in water). Thus, in the case of co-precipitation, the inorganic alkaline solution (NaOH/Na_2_CO_3_) was replaced by an organic one of TMAH while all the synthesis steps and tools were maintained identical. The synthesized materials were denoted as **LDH-TMAH-CP**, **cLDH-TMAH-CP**, and **hyLDH-TMAH-CP**, respectively. For the mechano-chemical route, a volume of TMAH equal to the one employed in the co-precipitation was used, while maintaining all the other procedures identical to those employed for **LDH-CO_3_^2−^/OH^−^-MC**. The initial sample and its derivatives were denoted as **LDH-TMAH-MC**, **cLDH-TMAH-MC**, and **hyLDH-TMAH-MC**, respectively. In the presence of TMAH the processes occurred with a significant decrease (10 times less) of bi-distilled water consumption in the washing step compared to the syntheses in the presence of inorganic alkalis.

### 3.2. Catalyst Characterization

Solid powder XRD diffraction patterns were recorded on a Shimadzu XRD 7000 diffractometer with Cu Kα radiation (λ = 1.5418 Å, 40 kV, 40 mA) at a scanning speed of 0.10°·min^−1^ using the 2θ range between 5–80°. DRIFTS spectra were recorded with JASCO FT/IR-4700 spectrometer by acquisition of 128 scans in 4000–400 cm^−1^ domain. DR UV-VIS spectra were recorded in the range 900–200 nm on Jasco V-650 UV-VIS spectrophotometer equipped with an integration sphere and using Spectralon as white reference. Raman spectra were collected with a Horiba Jobin Yvon–Labram HR UV–Visible–NIR Raman Microscope Spectrometer, using a 632 nm laser. The spectra were collected at the average of 10 scans at a resolution of 2 cm^−1^ between 100–4000 cm^−1^ Raman Shift. N_2_ adsorption-desorption isotherms were determined using a Micromeritics ASAP 2010 instrument, where prior to N_2_ adsorption-desorption, a vacuum for 24 h at 120 °C was involved for samples degassing. The total number of base sites in the catalysts was determined by the irreversible adsorption of acrylic acid (*pKa* = 4.2) [62,63,64].

### 3.3. Catalytic Tests

#### 3.3.1. Claisen–Schmidt Condensation

Claisen–Schmidt condensation was performed in a thermo-stated glass reactor equipped with a water cooled condenser, where a mixture of cyclohexanone (0.001 mol, >99%, Sigma–Aldrich (St. Louis, MO, USA)), benzaldehyde (0.002 mol, >99%, Sigma–Aldrich) and 20 mg of catalyst was stirred (600 rpm) under solvent-free conditions for 2 h at 120 °C [41]. After that, the catalyst was removed by filtration, washed with 1 mL of toluene, and the liquid mixture was analyzed by Thermo-Quest GC provided with a FID detector and a capillary column of 30 m length with DB5 stationary phase. The compounds were also identified using a GC/MS/MS Varian Saturn 2100 T equipped with a CP-SIL 8 CB Low Bleed/MS column of 30 m length and 0.25 mm diameter.

#### 3.3.2. The Aldol Cyclohexanone Self-Condensation

The cyclohexanone self-condensation was carried out in a reactor similar to the above-described one by mixing 0.01 mol of cyclohexanone with 50 mg catalyst under solvent-free conditions [47]. After 2 h at 120 °C, the catalyst was removed from the mixture by filtration and the liquid reaction mixture was analyzed by GC-FID. Furthermore, GC/MS was used for compound identification.

#### 3.3.3. Benzaldehyde Oxidation

The benzaldehyde oxidation by air was carried out under ambient pressure in the same reactor taking 0.01 moles of benzaldehyde and 53 mg catalyst under solvent-free conditions. After 2 h at 120 °C, the catalyst was removed from the mixture by filtration and the reaction mixture was analyzed by GC-FID. Furthermore, GC/MS was used for the compound identification.

### 3.4. Catalyst Recycling

The catalytic activity of mixed oxides was evaluated in 3 successive tests. The catalysts were separated from the reaction mixture by filtration, washed with 1 mL of toluene, and dried for 5 h at 120 °C in air before subsequent use.

## 4. Conclusions

The synthesized Fe-LDH materials contained Fe_3_O_4_ regardless of the preparation method or alkalis used in the syntheses. The highest crystallinity was displayed by LDH-TMAH-CP, whereas LDH-TMAH-MC exhibited the lowest one. The hydroxyl/hydroxycarbonate phases of Fe^2+^ and Fe^3+^ were encapsulated in an amorphous form in the parent LDH. FeO, Fe_2_O_3_, and Fe_3_O_4_ were also present in the sample prepared by the mechano-chemical method with TMAH. The catalysts basicity paralleled the conversion of cyclohexanone in the Claisen–Schmidt condensation, i.e., mixed oxides > parent LDH > hydrated LDH. The calcination of LDH provided an enhanced catalytic activity (93% conversion) for a total selectivity to 2,6-dibenzylidenecyclohexanone. The self-condensation of cyclohexanone is directed towards the *mono*-condensation product, 2-(1-cyclohexenyl)-cyclohexanone at a maximal conversion of 3.8% (cLDH-TMAH-CP). The presence of a single or double bond between the fragments of the *mono*-condensed product, corroborated with the size of the pores in the catalyst, are the essential factors in orientating the transformation.

## Figures and Tables

**Table 1:** The structural parameters of the prepared samples.

Hydrotalcite Samples	Lattice Parameters		IFS ^1^ (Å)	2θ*_003_* (°)	I*_003_*/I*_006_*	I*_003_*/I*_110_*	FWHM*_003_*	D ^2^ (Å)
	*a* (Å)	*c* (Å)						
LDH-CO_3_^2−^/OH^−^-CP	3.004	22.039	2.55	12.6607	4.30	2.48	0.3120	256
hyLDH-CO_3_^2−^/OH^−^-CP	3.036	22.412	2.67	11.8866	1.70	1.42	0.2667	300
LDH-CO_3_^2−^/OH^−^-MC	3.040	22.492	2.70	11.9400	3.50	2.80	0.4400	182
hyLDH-CO_3_^2−^/OH^−^-MC	3.041	23.029	2.88	11.4720	1.50	1.17	0.1040	667
LDH-TMAH-CP	3.034	22.575	2.72	11.7720	3.73	4.97	0.7200	111
hyLDH-TMAH-CP	3.033	23.035	2.88	11.5400	0.57	0.57	0.1200	665
LDH-TMAH-MC	3.038	22.920	2.84	11.2666	1.13	1.38	0.1333	600
hyLDH-TMAH-MC	3.033	22.283	2.63	11.9000	0.64	1.00	0.0534	1296
**Mixed oxide samples**	***a* (Å)**			**2θ*_311_* (°)**	**I*_311_***		**FWHM*_311_***	**D ^3^ (Å)**
cLDH-CO_3_^2−^/OH^−^-CP	4.995			35.9143	59		0.2087	400
cLDH-CO_3_^2−^/OH^−^-MC	5.025			35.7083	60		0.7967	105
cLDH-TMAH-CP	5.021			35.7333	95		0.7333	114
cLDH-TMAH-MC	4.998			35.9074	123		0.4709	177

^1^ IFS represents the interlayer free distance calculated from the network parameter ***c*** in which 4.8 Ǻ is brucite sheet thickness [51]. ^2^ D represents the mean crystallite size (derived from the Debye–Scherrer equation) determined from the FWHM of the *003* reflection for LDH samples. ^3^ D represents the mean crystallite size (derived from the Debye–Scherrer equation) determined from the FWHM of the *101* reflection for mixed oxides.

**Table 2:** The surface area and basicity of catalysts.

Samples	Surface Area (m^2^·g^−1^)	Pore Volume (cm^3^·g^−1^)	Average Pore Width (Å)	Total Number of Base Sites (mmol·g^−1^) ^1^
LDH-CO_3_^2−^/OH^−^-CP	103	0.505	191	7.21
cLDH-CO_3_^2−^/OH^−^-CP	263	0.831	127	7.23
hyLDH-CO_3_^2−^/OH^−^-CP	37	0.199	209	7.15
LDH-CO_3_^2−^/OH^−^-MC	170	0.503	113	7.14
cLDH-CO_3_^2−^/OH^−^-MC	274	0.875	103	7.25
hyLDH-CO_3_^2−^/OH^−^-MC	42	0.213	222	6.96
LDH-TMAH-CP	69	0.217	120	7.16
cLDH-TMAH-CP	223	0.678	131	7.20
hyLDH-TMAH-CP	101	0.587	183	6.88
LDH-TMAH-MC	154	0.177	43	6.85
cLDH-TMAH-MC	231	0.764	139	7.15
hyLDH-TMAH-MC	139	0.438	111	6.73

^1^ mmol of acrylic acid.

**Table 3:** The benzaldehyde conversion in oxidation reaction with molecular oxygen from air under ambient pressure (2 h, 120 °C, 53 mg of catalyst, solvent-free).

Catalysts	Benzaldehyde Conversion (%)
blank	31.5
LDH-CO_3_^2−^/OH^−^-CP	0.5
cLDH-CO_3_^2−^/OH^−^-CP	0.6
hyLDH-CO_3_^2−^/OH^−^-CP	0.2
LDH-CO_3_^2−^/OH^−^-MC	0.4
cLDH-CO_3_^2−^/OH^−^-MC	4.5
hyLDH-CO_3_^2−^/OH^−^-MC	4.5
LDH-TMAH-CP	3.4
cLDH-TMAH-CP	4.8
hyLDH-TMAH-CP	3.4
LDH-TMAH-MC	2.4
cLDH-TMAH-MC	6.0
hyLDH-TMAH-MC	3.5

**Table 4:** Experimental data for self-cyclohexanone conversion (2 h, 120 °C, 50 mg catalyst, solvent-free).

Catalysts	Conv. C_6_H_10_O (%)	Sel. (%) Mono-A	Sel. (%) Mono-B	Sel. (%) Di-A	Sel. (%) Di-B
LDH-CO_3_^2−^/OH^−^-CP	0.8	60.1	37.8	1.1	1.0
cLDH-CO_3_^2−^/OH^−^-CP	0.9	71.0	28.8	0.1	0.1
hyLDH-CO_3_^2−^/OH^−^-CP	0.8	62.2	37.2	0.5	0.1
LDH-CO_3_^2−^/OH^−^-MC	0.3	63.3	36.7	0.0	0.0
cLDH-CO_3_^2−^/OH^−^-MC	0.7	78.5	21.5	0.0	0.0
hyLDH-CO_3_^2−^/OH^−^-MC	0.5	70.8	29.2	0.0	0.0
LDH-TMAH-CP	1.2	75.6	13.0	5.5	5.9
cLDH-TMAH-CP	3.8	83.9	14.5	1.4	0.2
hyLDH-TMAH-CP	3.3	77.0	21.7	1.1	0.2
LDH-TMAH-MC	0.6	78.0	22.0	0.0	0.0
cLDH-TMAH-MC	1.0	81.1	18.9	0.0	0.0
hyLDH-TMAH-MC	0.9	80.0	20.0	0.0	0.0

## Data Availability

The data are available on request from the corresponding authors.

## Sample Availability

Samples of the compounds LDH-CO_3_^2−^/OH^−^-CP; LDH-CO_3_^2−^/OH^−^-MC; LDH-TMAH-CP and LDH-TMAH-MC are available from the authors.
